# Recent Advances in the Properties and Applications of Polyglycerol Fatty Acid Esters

**DOI:** 10.3390/polym17070879

**Published:** 2025-03-25

**Authors:** Mingyu Zhang, Guangyan Zhang

**Affiliations:** School of Materials and Chemical Engineering, Hubei University of Technology, Wuhan 430068, China; zhangmingyu337@163.com

**Keywords:** polyglycerol, polyglycerol fatty acid ester, PGFEs, non-ionic surfactants, emulsion, HLB

## Abstract

Although polyethylene glycol (PEG)-based surfactants are widely used in various industries due to their wide range of hydrophile–lipophile balance (HLB) values, their possible by-product, 1,4-dioxane, has been listed as a reasonably anticipated human carcinogen, which may limit their applications in fields closely related to the human body. Polyglycerol fatty acid esters (PGFEs), a class of surfactants based on polyglycerol (another polyether), also have a wide range of HLB values that can be adjust by varying the degree of polymerization of the polyglycerol, the length of the fatty acid carbon chain, or the degree of esterification, but do not have the risks caused by 1,4-dioxane. In addition, all the raw materials (glycerol and fatty acids) required for the preparation of PGFEs can be obtained via hydrolysis of renewable vegetable oils. Therefore, PGFEs, as new eco-friendly and biodegradable non-ionic surfactants, have been proposed as potential green alternatives to PEG-based non-ionic surfactants. This review summarizes the properties of PGFEs specifically including their HLB, surface properties, phase behaviors, stabilizing effect on foams and emulsions, and stability, and highlights their potential applications in food, cosmetics, and pharmaceuticals observed in the last decade.

## 1. Introduction

Surfactants are amphiphilic organic molecules, usually composed of a hydrophobic “tail” and a hydrophilic “head” linked by various covalent bonds. The hydrophobic “tail” can be aromatic group or alkyl chains containing 8–22 carbons, and the hydrophilic “head” usually includes polar moieties (e.g., a hydroxyl group and ether bond) or ionic moieties (e.g., carboxylate and sulfonate). Surfactants are usually divided into non-ionic, anionic, cationic, and amphoteric types based on the composition of their hydrophilic heads. Among them, non-ionic surfactants, including polyethylene glycol (PEG)-based surfactants [1,2], glycerol-based surfactants [3,4], and sugar-based surfactants [5,6], have attracted widespread attention in the fields of cosmetics, food, and pharmaceuticals due to their lower toxicity and reduced irritant effects compared to ionic surfactants [7].

PEG-based surfactants, one of the most important non-ionic surfactants, include alkyl PEG ethers, PEG fatty acid esters, block copolymers of PEG and polypropylene glycol (PPG) (e.g., poloxamers 184), and alkyl PEG/PPG ethers. However, during the manufacturing process of PEG-based surfactants, 1,4-dioxane may be generated as a by-product, which is reasonably anticipated to be a human carcinogen (the 15th Report on Carcinogens) and unsafe for the human body [8]. Moreover, PEG is a synthetic polyether compound derived from petroleum (a non-renewable resource), and its wide-scale use in the preparation of surfactants does not meet the needs of sustainable development. Polyglycerol (PG) is a polyether that can be polymerized from glycerol obtained through the hydrolysis of renewable vegetable oils. Polyglycerol also can be used as the hydrophilic part of surfactants, replacing PEG in the preparation of non-ionic surfactants, with no potential risk caused by 1,4-dioxane. Therefore, with the increasing awareness of the importance of health and sustainable development, polyglycerol-based non-ionic surfactants have attracted extensive attention.

Polyglycerol fatty acid esters (PGFEs) are the most extensively investigated polyglycerol-based non-ionic surfactants because the raw materials (e.g., glycerol and fatty acids) required to prepare PGFEs can be derived from renewable vegetable oils. Glycerol-based non-ionic surfactants are also renewable surfactants, but they are usually used as water-in-oil (w/o) emulsifiers due to their low hydrophile–lipophile balance (HLB) [3]. Sugar-based non-ionic surfactants, including sorbitan esters, alkyl polyglucosides, and sucrose esters, are another type of renewable surfactants that have been extensively studied [9]. However, the typical HLB value of sorbitan esters is usually in the range of 1–8, which is mainly suitable for oil-based systems [9], while the HLB value of alkyl polyglycosides is usually above 9, making it mainly suitable for water-based systems [10]. Compared with glycerol-based surfactants, sorbitan esters, and alkyl polyglucosides, PGFEs can be used not only in oil-based systems but also in water-based systems, because high lipophilicity (a low HLB value) can be obtained by decreasing the degree of polymerization of PG or increasing the degree of esterification, while high hydrophilicity (high HLB value) can be obtained by increasing the degree of polymerization of PG or decreasing the degree of esterification.

Although sucrose esters are also suitable for both oil-based and water-based systems due to their wide HLB value range of 1–16 [5,11], the esterification of sucrose requires perfect control due to sucrose’s sensitivity to temperature. In addition, the use of harmful DMSO and DMF as solvents in the production process of sucrose esters is of great concern [9]. Compared with the production of sucrose esters, the production of PGFEs does not require the use of toxic organic solvents, and PG is not sensitive to temperature, which makes it very attractive.

**Scheme 1:**
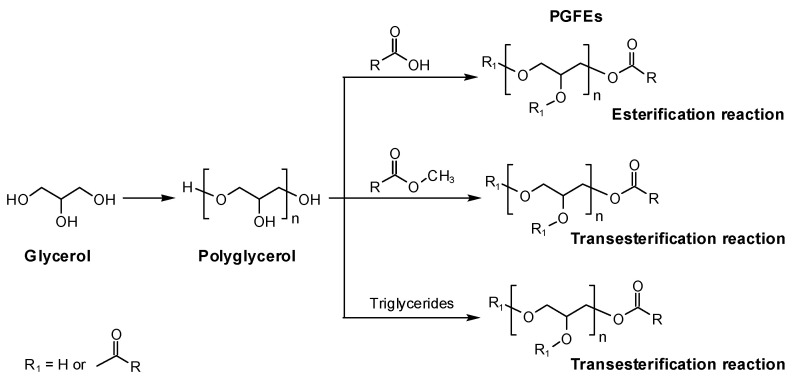
The typical synthesis routes of polyglycerol fatty acid esters.

The typical preparation of PGFEs is as follows: (1) glycerol is dehydrated and polymerized using alkali (e.g., NaOH and K_2_CO_3_) or acid (e.g., H_2_SO_4_) as a catalyst to prepare polyglycerol, and the average degree of the polymerization of PG can be regulated via controlling the dehydration; (2) then, PGFEs can be prepared using (a) the esterification reaction between PG and fatty acids or (b) the transesterification reaction of PG with fatty acid esters or triglycerides in the absence of solvents (Scheme 1).

**Figure 1:**
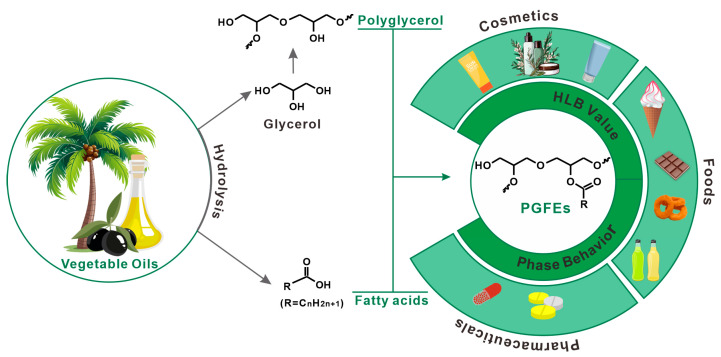
The typical preparation route and applications of polyglycerol fatty acid esters.

PGFEs are not only eco-friendly and free of 1,4-dioxane during preparation, but they also have outstanding application performances in various fields (Figure 1). In the food field, PGFEs have been approved as food additives (E475) [12] in many countries, and are mainly used as emulsifiers and crystallization-adjusters. In the cosmetic field, PGFEs have been used as emulsifiers for the preparation of moisturizing creams or as detergents for the preparation of cleaning products. In the pharmaceutical field, PGFEs have been investigated for their use as lipid-based excipients. Moreover, the cost of PGFEs is comparable to that of glycerol-based surfactants and sugar-based surfactants [13], and this cost can be further reduced when they are prepared via transesterification with vegetable oils [14].

Although the synthesis and methods of analysis of PGFEs have been summarized [15], their applications and related important properties (e.g., HLB value and phase behavior) have not been reviewed in detail in recent years. In this review, the properties of PGFEs, including their HLB values and phase behaviors in various mixture systems, are summarized, with an emphasis on their potential applications in food, cosmetics, and pharmaceuticals observed in the last decade.

## 2. Properties of PGFEs

The hydrophile–lipophile balance (HLB) is one of the most important parameters when evaluating the suitability of a surfactant for a specific application, as it reflects the partitioning tendency of a surfactant between oil and water. In addition to the HLB value, the phase behavior also plays a significant role in the practical application of surfactant mixtures. This not only reflects how surfactant molecules aggregate in a specific surfactant system, but is also closely related to physical properties such as viscosity and rheological properties.

### 2.1. Hydrophile–Lipophile Balance

The HLB value was first proposed by Griffin and generally ranges from 0 to 20 [16]. For non-ionic surfactants, the HLB value is defined as the hydrophilicity to hydrophobicity ratio of a surfactant, and can be calculated using Equation (1):

(1) $$\mathrm{HLB}=20\;\text{×}\;\frac{M_{h}}{M_{t}}=20\;\text{×}\;\frac{S}{A}$$

where M_h_ is the molecular weight of the hydrophilic moiety of the surfactant, M_t_ is the molecular weight of the entire surfactant, S is the saponification value of the surfactant, and A is the acid value of the surfactant. Davies’ group numbers contribution method is another commonly used method to estimate the HLB value of surfactants, as shown in Equation (2).

(2) HLB=7+∑hydrophilic group contribution−∑lipophilic group contribution

In addition to the methods of Griffin and Davies, the HLB values of PGFEs also can be estimated from organic conceptual diagrams [17] via converting the inorganic/organic balance (IOB) value to the HLB value with Equation (3) [18].

(3) HLB=10 × IOB

Based on the methods used to estimate HLB value, the HLB values of PGFEs increase with an increase in the degree of polymerization of the polyglycerol (hydrophilic moiety), and decrease with an increase in the length of the fatty acid (FA) carbon chain (hydrophobic moiety) or the degree of esterification of PGFEs. The chemical structures, HLB values and uses of investigated PGFEs are summarized in Table 1. As shown in Table 1, similarly to PEG-based surfactants, the HLB values of PGFEs can be adjusted in a wide range from 1.4 to 19.0.

For practical purposes, the HLB value is extensively used in the design of formulations of water/oil systems in surfactants. Generally, surfactants with a range of HLB values, e.g., 3–6, can be used to prepare water-in-oil (w/o) emulsions, while surfactants with an HLB range of 8–18 can be used to prepare oil-in-water (o/w) emulsions [16]. As shown in Table 1, PGFEs are most commonly studied for their use as emulsifiers. Regarding emulsification, a series of PG-5 fatty acid esters with HLB values ranging from 1.4 to 16.6 were designed and synthesized to investigate the relationship between emulsifying properties and HLB. The results showed that when the oil–water ratio was 1:1, PG-5 fatty acid esters with HLB values of greater than nine formed o/w emulsions, while those with HLB values less than nine formed w/o emulsions [21]. Of course, the type of PGFEs used as emulsifiers depends not only on the HLB value, but also on the oil-–water ratio of the formulation system. In addition to emulsification, PGFEs with low HLB values in the range of 1.6–6.0 can be used as crystallization-adjusters [23,24,25] and lipid-based excipients [26] due to their good lipophilicity; some PGFEs with high HLB values of around 14 have been reported to be applied as detergents [37]. The detailed applications of PGFEs will be further discussed in Section 3.

### 2.2. Surface Properties of PGFEs

Changes in the hydrophilic “head” (PG) and hydrophobic “tail” (fatty acid) not only alter the HLB values of the surfactants, but also alter their surface properties, including their critical micelle concentration (CMC), surface tension, and interfacial tension (Table 2).

As shown in Table 2, the CMC of PGFEs usually increases with an increase in the average degree of polymerization of PG moieties and decreases with an increase in the aliphatic chain length or the degree of esterification. PGFEs can effectively reduce the surface tension of water (about 72 mN/m). The surface tension value of most PGFEs at CMC reported in the literature was around 30 mN/m, and a few PGFEs (PG-2 monolaurate [38], PG-2 monooleate [39], and PG-10 mono-caprylate/caprate [22,40]) can reduce the surface tension of water to 27 mN/m.

**Table 2:** Surface properties of PGFEs.

PGFEs	CMC (mg/L)	γ_CMC_ (mN/m)	Interfacial Tension (mN/m)	Ref.
PG-2 monolaurate	36.54	27.7	5.5 ^a^	[38]
PG-2 monooleate	0.12 mmol/L	27	5.5	[39]
PG-2 stearate (Enzymatic or chemical method)	-	-	~2 ^b^ (0.4 g/L)	[41]
PG-2 monoester	39.09	31.96	4.81 ^c^	[14]
PG-2 diester	28.12	33.54	7.22 ^c^	[14]
PG-2 triester	15.80	35.99	7.45 ^c^	[14]
PG-3 monoester	69.70	31.55	4.99 ^c^	[14]
PG-3 diester	53.22	33.89	5.80 ^c^	[14]
PG-3 triester	31.04	35.76	6.35 ^c^	[14]
PG-3 monolaurate	78.91	31.4	1.7 ^a^	[38]
PG-4 monolaurate	118.05	35.2	3.4 ^a^	[38]
PG-5 monolaurate	168.15	39.6	5.3 ^a^	[38]
PG-10 mono-caprylate/caprate	10–30	27.13	14 ^b^	[22,40]
PG-10 monolaurate	30	~29	20 ^b^	[22]
PG-10 monostearate	-	-	25 ^b^	[22]

^a^ corn oil/aqueous solution of PGFEs (corn oil/water: 23.9 mN/m), 30 °C; ^b^ sunflower oil/aqueous solution of PGFEs (sunflower oil/water: 31 mN/m); ^c^ n-heptane/aqueous solution of PGFEs (n-heptane/water: 48.5 mN/m). “-” = Not provided.

In addition, a notable reduction in the interfacial tensions (IFT) in various oil/water systems was also observed. It is worth noting that the purified PG-3 monolaurate significantly reduced the interfacial tension of corn oil/water from 23.9 mN/m to 1.7 mN/m at a concentration of 0.1% [38]. Another study revealed that as the concentration of PG-2 stearate increased, the interfacial tension of the sunflower oil/water system decreased from approximately 20 mN/m (0.04 g/L) to approximately 2 mN/m (0.4 g/L). Interestingly, although both chemically and enzymatically synthesized PG-2 stearate showed a similar minimum interfacial tension (around 2 mN/m), chemically synthesized PG-2 stearate showed a constant IFT decrease from 0.25 g/L, while enzymatically synthesized PG-2 stearates showed an abrupt decrease at 0.37 g/L [41]. This may be due to the fact that the composition of enzymatically synthesized PG-2 stearate (PG-2: 6%; PG-2 stearate: 90%; other: 4%) is relatively simple compared to that of chemically synthesized PG-2 stearate (PG-2: 5%; PG-2 stearate: 70%; PG-3 stearate + PG-4 stearate + other: 25%), which was ascribed to the higher selectivity and fewer side reactions of enzymatic synthesis. The PG-3 stearate and PG-4 stearate found in chemically synthesized PG-2 stearate may be attributed to the further polymerization of PG-2.

### 2.3. Phase Behaviors of Various Systems Containing PGFEs

Although the HLB value of a surfactant is a crucial and effective parameter for its practical applications, its phase behavior also plays an important role in practice because this can reflect the arrangement of surfactant molecules in a system or at an interface, which generally has a great influence on the viscosity [42], rheological properties [43], and stability of emulsions or foams [44]. For example, in the same mixture system, the viscosity of the micellar phase is usually significantly lower than that of the liquid crystal phase, and the formation of various phases depends on their different compositions. Therefore, the phase behaviors of various mixture systems containing PGFEs are worthy of attention and are summarized in Table 3.

Hashizaki et al. investigated the effects of PGFEs on the lecithin/n-decane system, and found that PGFEs can induce the formation of a long cylindrical reverse worm-like micellar phase (PGFE/lecithin/n-decane) from the spherical or ellipsoidal reverse micellar phase (lecithin/n-decane). Due to the addition of PGFEs, the lecithin/n-decane solution was transformed into a highly viscoelastic solution composed of reverse wormlike micelles and exhibited the rheological property of “shear-thickening”. In addition, with the increase in PGFEs concentration, the number and length of the reverse worm-like micelles increased, resulting in a significant increase in the zero-shear-rate viscosity (η_0_) of PGFE/lecithin/n-decane solution [18]. Additionally, lamellar gels formed by PG-10 monostearate/cetyl isooctanoate/water ternary system also can be used to prepare o/w emulsions with a high dispersion stability just by diluting them with water (low-energy emulsification method) [45].

**Table 3:** The phases of mixed systems containing PGFEs.

PGFEs	Systems	Phases ^1^	Ref.
PGL6FA10	PGFE/lecithin/n-decane	Om	[18]
PGL10FA6		Om	
PGL10FA10		Om	
PGL10FA14		Om	
PGL10FA18		-	
PGL20FA10		Om	
PGL40FA10		Om	
PG-10 monolaurate	PGFE/ethanol/water	lamellar gel	[31]
PG-10 monomyristate			
PG-10 monopalmitate			
PG-10 monostearate			
PG-2 monostearate			
PG-10 monostearate	PGFE/cetyl isooctanoate/water	Lα	[45]
PG-x didodecanoates (x = 4.5, 7.3, 10.7, 21.3)	PGFE/water	Lα, Lα + W, H_1_, Wm	[46]
PG-5 monostearate	PGFE/water	Lα + W, Lα (>46 °C),	[47]
		α-gel + W, α-gel (<46 °C)	
PG-5 monooleate	PGFE/water	Lα + W, Lα, Om	
PGE 55	PGFE/water	MLV + W, Lα (>58 °C) MLV + α-gel, α-gel (<58 °C)	[48,49]
SY-Glyster ML-750 (PG-10 monolaurate)	(PGFE/polyglycerol polyricinoleate)/	L_3_, Lc + L_3_, L_3_ + Wm + O, Wm + O	[50]
SY-Glyster ML-550 (PG-6 monolaurate)	water/vegetable oil	Lα + L_3_, Lc + L_3_, L_3_ + Wm + O, Wm + O	
SY-Glyster MM-750 (PG-10 monomyristate)	PGFE/polyglycerol polyricinoleate = 1:1	L_3_, Lc + L_3_, L_3_ + Wm + O, Wm + O	
SY-Glyster ML-550 (PG-6 monolaurate)	(PGFE/polyglycerol polyricinoleate)/ glycerol/vegetable oil PGFE/polyglycerol polyricinoleate = 1:1	Lc + L_3_ + G, Lc + L_3_, Lc + L_3_ + O, Lc + L_3_ + G + O	[51]

^1^ Om = reverse micellar phase; Lα = lamellar phase; W = isotropic aqueous phase; H_1_ = normal hexagonal phase; Wm = normal micellar phase; MLV = multilamellar vesicles; L_3_ = sponge phase; Lc = liquid crystalline phase; O = oil phase; G = glycerol phase. “-” = Not observed.

### 2.4. Stabilizing Effect on Foams and Emulsions

Foam is an important sensory indicator of certain products. Therefore, foaming ability and foam stability are critical for surfactant applications. The initial foam heights of 0.1% (1%) PG-10 mono-caprylate/caprate, PG-10 monolaurate, PG-10 monostearate and PG-10 monooleate were 22 mm (56 mm), 17 mm (44 mm), 5 mm (36 mm), and negligible (2 mm), respectively (stirring method), indicating that when PG-10 is used as the hydrophilic head, the shorter the fatty chain, the better the foaming ability [22]. Although the initial foam heights of the PGFEs aqueous solution were relatively low, the foam heights did not change much over time [22,38], indicating that the foam formed by PGFE has good stability. In addition, PGE 55, a commercial PGFE prepared from oligoglycerol (the average degree of polymerization is about 3) is an efficient surfactant for foam generation that can generate a large amount of foam with very small bubble size (about 7 mg surfactant for 1 m^2^ surface area) [28]. Another report showed that pH has a significant impact on foam volume. At a pH of 3, nearly 3 L foam formed in just 60 s, while at a pH of 9, only 100 mL foam was produced after 5 min of whipping [52].

To maintain the stability of foams and emulsions, interfacial film is one of the key factors. Taking the o/w emulsion as an example, the more rigid the interfacial film is the greater the amount of energy required to destroy it. Thus, the more difficult it is for the emulsion to coalesce, the more stable the emulsion. Adding surfactants that can form a lamellar phase is an effective way to improve the stability of emulsions because the ordered lamellar structure can enhance the rigidity of the interfacial film.

For instance, Sakai et al. prepared ethanol-containing o/w emulsions (ethanol content: 40 wt.%) with long-term stability (more than 4 months) using a mixture of PG-10 monostearate and PG-2 monostearate as an emulsifier. The good stability was attributed to the adsorption of the lamellar phase at the o/w interface, forming a rigid interfacial film with excellent viscoelastic properties [31].

### 2.5. Stability of PGFEs

The thermal stability of PGFEs under neutral conditions is relatively good and can be considered stable below 100 °C [53]. When the storage temperature exceeds 100 °C, the instability will be enhanced. In terms of hydrolysis, PGFEs are comparable to monoglycerides [54]. Their hydrolysis will be accelerated in the presence of bases or acids due to the ester linkage between PG and fatty acids. In addition, similarly to other glycerides, PGFEs can also be degraded by lipases [55].

## 3. Applications of PGFEs

Since the raw materials (e.g., glycerol and fatty acids) for the preparation of PGFEs are derived from natural sources, they are considered to possess high bio-safety and are approved as food additives [53] and cosmetic ingredients [55]. Their safety as pharmaceutical excipients has been evaluated in recent years [56].

### 3.1. Food Field

In the food field, fats and oils are important ingredients. The crystallization behaviors of fats and oils used in food products has a strong influence on the overall structures of fat-/oil-containing products and ultimately affects the physical properties of the final products.

#### 3.1.1. Oils and Fats

Palm oil, an edible oil derived from palm fruit, exhibits a semi-solid state at room temperature. It can be fractionated into a high-melting solid fraction (palm stearin) and a low-melting liquid fraction (palm olein) [57]. The quality of the two fractions is related to the crystallization and separation steps. For the crystallization step, PGFEs can be used as a crystallization-adjuster to retard crystal growth. For example, PGEmix-8 (a polyglycerol mixed-fatty acid ester with melting point of 42.8 °C) can promote nucleation and retard crystal growth, thereby significantly affecting the crystallization process of palm oil and reducing the fractionation time [24,25]. Interestingly, the effects of PGFEs on the crystallization behavior of palm olein are not consistent. Palsgaard^®^ PGE 1105 and PGE 1117 decreased the crystallization induction time and increased the crystallization rate of palm olein, while Palsgaard^®^ PGE1155 significantly decreased the crystallization rate [58].

Oils rich in diacylglycerols (DAG) are a promising alternative to conventional edible oils in healthy daily diets because DAG have health benefits such as reducing serum triacylglycerol levels and suppressing body weight and the amount of visceral fat. However, compared with triglyceride-based oils, DAG-rich oils are more susceptible to precipitation at low temperatures, limiting their applications. According to the literature, different PGFEs had different effects on DAG crystallization. Commercial PG-10 oleate (Q-1710) did not retard the crystallization of DAG-rich oils, whereas polyglycerol fatty acid ester, which was fully esterified with a mixture of palmitic acid/oleic acid (50/50) and PG-10, significantly retarded the crystallization of DAG-rich oils [59,60]. In addition to PG-10 fatty acid esters, PG-2 linoleates also can retard the crystallization of DAG-rich oil and the retardation effect increased as the esterification degree of PG-2 linoleates decreased [61].

PGFEs also can adjust the crystallization of fats. For example, the polyglycerol fatty acid ester, fully esterified with PG-3 and stearic acid, can effectively promote the nucleation of fat blend to form small and uniform crystals, thereby improving the quality of whipped cream [62]. From the results of the effects of various PGFEs on the crystallization of oils and fats, the chemical structure of PGFEs, such as the type and composition of fatty acids and the degree of esterification of hydrophilic polyglycerol, may be an important factor regulating the crystallization.

In addition to adjusting the crystallization, PGFEs (e.g., polyglycerol stearate and polyglycerol behenate) have also been reported to act as oleogelators [63,64,65] in the structure of liquid oils (e.g., soybean oil [66] and sunflower oil [67]) or low-saturated fat blends [68], or to act as antioxidants to inhibit the lipid oxidation and improve the shelf life of soybean oil [69].

#### 3.1.2. Bioactive Ingredients

Bioactive ingredients such as polyphenols provide some potential health benefits, but adding bioactive ingredients to foods to enhance their functionality leads to many challenges. How to improve the stability and bioaccessibility of bioactive ingredients in food has always been an urgent problem to be solved.

Curcumin (CUR) is a polyphenol that has attracted much attention in the development of functional foods due to its antioxidant, anti-inflammatory, and other physiological activities. However, CUR is highly hydrophobic with a very low oral bioavailability of less than 0.5%. In order to improve the water-solubility of CUR, PGFEs can be used to prepare CUR-loaded emulsions [27,70] or enhance the stability of CUR-loaded emulsions [71,72]. For example, Zhang et al. prepared CUR-loaded nano-emulsions stabilized by PGFEs and investigated the effect of the aliphatic chain length in PGFEs on the digestive profiles using an in vitro gastrointestinal tract (GIT) model [73]. Moreover, Nagano et al. reported an amorphous CUR formulation (CUR/polyvinylpyrrolidone (PVP)/PGFE = 11:64:25, *w*/*w*) with high and stable water-solubility using PGFE as an emulsifier, and revealed that PGFE can reduce the interaction between CUR, thereby improving the dispersibility and solubility of CUR in water [74].

In addition to CUR, lycopene also can be prepared in the nanodispersion using PGFE (M-7D) as an emulsifier. Interestingly, PGFEs can inhibit the conversion of the *Z*-isomer of lycopene to the all-*E*-isomer during storage [75]. Moreover, PGFEs have been reported to be effective emulsifiers for other lipophilic bioactive ingredients or nutrients, including co-enzyme Q10 [76], β-carotene [77], and peppermint oil [78].

#### 3.1.3. Other

In addition to the above applications in food, PGFEs also can be used to improve the quality of recombined dairy cream [29], increase the solubility of a flavor (e.g., lemon essential oil) to produce transparent flavor emulsions for beverages [79], or in combination with mono- and di-glycerides as a crystal-promoter to improve the quality of heat-resistant compound chocolates [80,81]. In addition, PG-3 monostearate was reported to be used as an additive to prepare stabilized 3D-printable capsanthin hydrogels for innovative foods [82], and polyglycerol monolaurates (PG-2 monolaurate, PG-3 monolaurate, and PG-6 monolaurate) were reported to exhibit antibacterial effects against Gram-positive bacteria, *S. aureus*, and *B. subtilis*, and may be utilized as a safe preservative in foods [83].

### 3.2. Cosmetic Field

PGFEs have become increasingly popular in cosmetic formulations as a potential alternative to PEG-based surfactants to avoid the risks caused by 1,4-dioxane, and have been used as emulsifying agents, emulsion stabilizers, cleansing agents, solubilizing agents, skin-conditioning agents, and hair-conditioning agents [55].

#### 3.2.1. Emulsifying Agent

**Figure 2:**
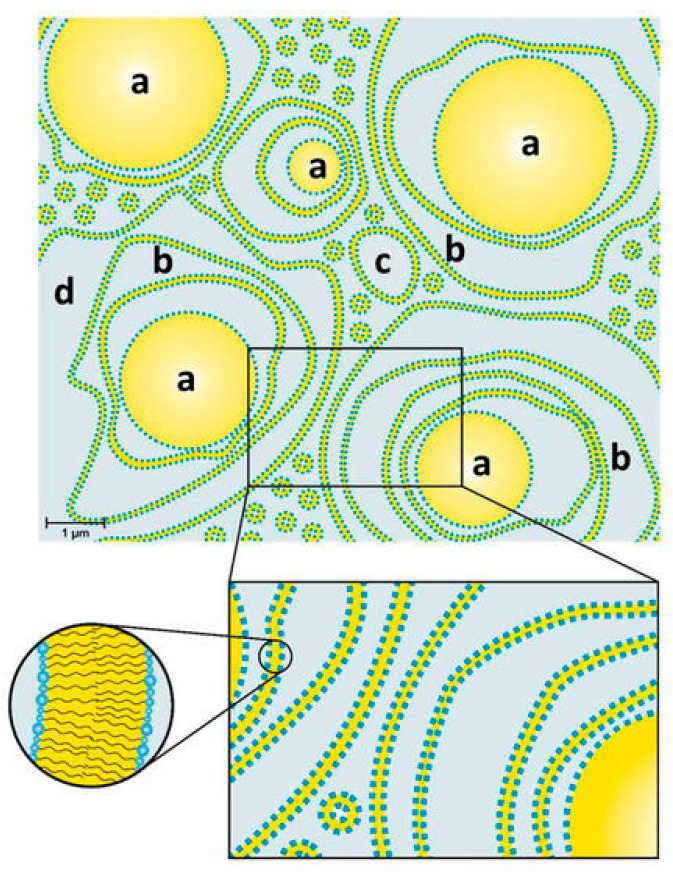
Model of an o/w-emulsion containing TEGO^®^ Care PSC 3 (an esterification product of PG-3 with stearic acid and a substoichiometric amount of citric acid) as emulsifier and glyceryl stearate/stearyl alcohol as consistency enhancer: (**a**) Oil droplets surrounded by (**b**) several layers of bilayers and (**c**) polydisperse vesicles in (**d**) a water phase [32].

Most cosmetic products come as emulsions. Although their uses are very different, their bases are very similar; they are primarily a mixture of water and oils (fats and lipids), along with some other ingredients. For example, Rajput et al. synthesized a series of PGFEs and used them as an emulsifying agent to successfully prepare moisturizing creams [14]. Dahl et al. investigated the structure of a typical cosmetic oil-in-water emulsion (o/w-emulsion) based on the emulsifier TEGO^®^ Care PSC 3 (an esterification product of PG-3 with stearic acid and a substoichiometric amount of citric acid) and the consistency enhancer (glyceryl stearate/stearyl alcohol). The results showed that pure TEGO^®^ Care PSC 3 forms lamellar stacked bilayers in water with a spacing of 7 nm, coexisting with polydisperse unilamellar vesicles. When the consistency enhancer was added, multilamellar vesicles could be obtained. In the final cosmetic o/w-emulsion with a cream-like consistency containing 25 wt.% oils, the oil droplets were surrounded by multiple irregularly spaced bilayers and vesicles, and about 30% of water was restricted in this system in the form of encapsulated water, as shown in Figure 2. This steric interaction between oil droplets and crystalline bilayers in water phase may be an important factor in enhancing the consistency of cosmetic emulsions [32].

In addition, PG-3 rice bran fatty acid esters have also been reported as being used as an emulsifier in sunscreen formulations containing UVA (butyl methoxydibenzoylmethane) and UVB (ethylhexyl methoxycinnamate) filters, and the protective effects of the sunscreen emulsions has been evaluated. Regarding the emulsions containing the UVA filter, the SPF value of the emulsion prepared with the PG-3 rice bran fatty acid esters was comparable to that of the emulsion prepared with the glucoside derivative Montanov 68 (cetearyl alcohol and cetearyl glucoside), but the decrease in the SPF value of the emulsion prepared with PG-3 rice bran fatty acid esters (9.62%) was clearly lower than that of Montanov 68 (12.28%), indicating that PG-3 rice bran fatty acid esters can improve UVA filters’ photostability [84].

#### 3.2.2. Cleansing Agent

Another use of PGFEs is as a cleaning agent. For instance, Iwanaga et al. successfully prepared an oil-based makeup remover that can be used even in a bath by utilizing the polyglycerol ester of middle-chain fatty acids [85]. For water-based makeup-removers, Zhang et al. investigated the aggregation behavior of PG-10 monocaprylate in water and the cleaning ability of its aqueous solution. The results showed that PG-10 monocaprylate could self-assemble into nanoparticles in the water with a transparent appearance (the concentration of PG-10 monocaprylate: 2.5–10 wt.%). Compared with the PEG-based non-ionic surfactants (e.g., PEG-6 caprylic/capric glycerides) used in transparent makeup removers, PG-10 monocaprylate is less irritating and exhibits a good removal effect against makeup, especially pen eyeliner [37]. It has been used as a sustainable alternative to PEG-6 caprylic/capric glycerides for the preparation of makeup-removers.

#### 3.2.3. Other

In addition to acting as an emulsifying agent and cleansing agent, PGFEs can also serve as a solubilizing agent to increase the solubility of red raspberry seed oil and/or hydro-glycolic acid fruit extract for the protection and hydration of skin [30]. The active ingredients (Bakuchiol and N-prolyl palmitoyl tripeptide-56 acetate) have also been reported to be incorporated into oil-in-water dermato-cosmetic emulsions using PG-6 stearate and PG-6 behenate, and the results showed that the obtained emulsions may be suitable for the preparation of cosmetics with potential anti-aging effects [86].

### 3.3. Pharmaceutical Field

In recent years, the application of PGFEs in the pharmaceutical industry has also received much attention. PGFEs can be used as lipid-based excipients, drug carriers, or for modifying nanomedicines.

#### 3.3.1. Lipid-Based Excipients

PGFEs were considered as an approach for overcoming the stability challenges faced by conventional lipid-based excipients, allowing for them to have advanced pharmaceutical applications due to their stable solid state, microstructure, and physicochemical properties [26,87,88]. For instance, ibuprofen-loaded inhalable lipid-microparticles were successfully prepared using PG behenates as lipid-based excipients via spray-drying [89]. Witepsol^®^ PMF 166 (PG-6 palmitate) can be used as a high-performing lipid material that is processed into blank filaments or felodipine-loaded filaments, and the obtained filaments can be further used to fabricate placebo dosage forms and personalized oral solid dosage forms via 3D-printing without using additional materials [90,91].

#### 3.3.2. Drug Carriers

**Figure 3:**
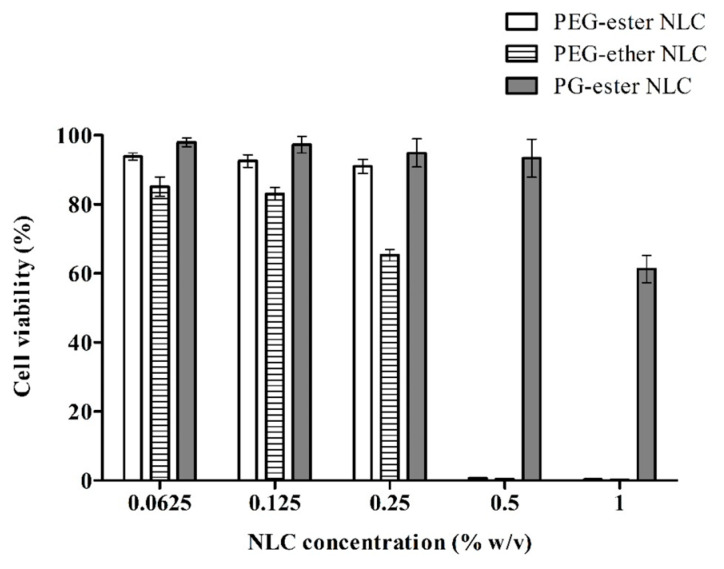
The viability of Caco-2 cells after 4 h incubation with insulin/sodium dodecyl sulfate complex-loaded NLCs, determined via resazurin assay [33].

In addition to lipid-based excipients, PGFEs can also be used as carriers for the delivery of proteins [92], peptides (e.g., insulin [33]), and hydrophobic drugs (e.g., cannabidiol [93]). For oral formulations, Bernkop-Schnürch’s group prepared three insulin/sodium dodecyl sulfate complex-loaded NLC (nanostructured lipid carrier) formulations containing PEG-ester, PEG-ether, and PGFE (TEGOSOFT^®^ PC 41 and TEGO^®^ Solve 90 MB) surfactants, respectively, and evaluated their cytotoxicity by resazurin assay against Caco-2 cells. The results showed that the non-cytotoxic concentration of PGFE-based NLC (0.5%) was significantly higher than that of PEG-based NLCs (PEG-ester NLC: 0.25%; PEG-ether NLC: 0.125%), as shown in Figure 3 [33].

**Figure 4:**
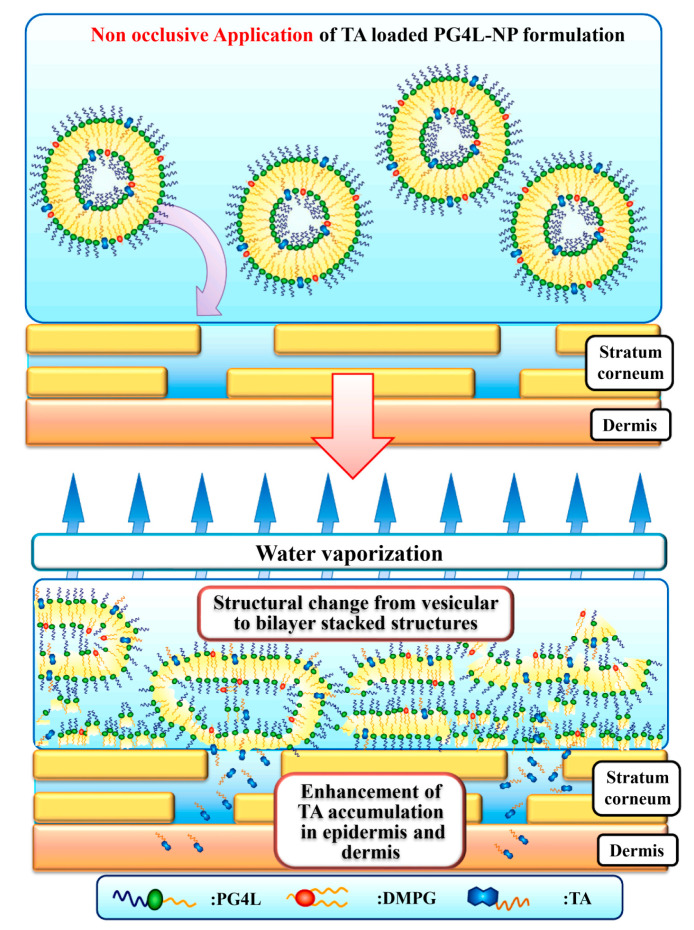
Speculated mechanism of TA accumulation from PG4L-NP under non-occlusive application. Reprinted from Ref. [35], Copyright (2021), with permission from Elsevier.

For topical applications, Uchino’s group developed PG-4 laurate-based tocopherol acetate-loaded nanoparticles (PG4L-NP) and evaluated the accumulation of tocopherol acetate (TA) in the epidermis and dermis. Interestingly, compared with other emulsifiers (e.g., soya phosphatidylcholine), only PG4L-NP induced TA accumulation in the dermis under non-occlusive applications. This may be due to the transformation of PG4L-NP from vesicles to bilayer stacked structures following water evaporation (Figure 4) [35].

#### 3.3.3. Other

PEGylation is a common strategy to enhance the stability, reduce the immunogenicity, and prolong the circulation time of protein drugs and nanomedicines (e.g., liposomes) [94,95]. However, the induced anti-PEG IgM production is thought to be associated with loss of therapeutic efficacy and increased adverse effects [96,97,98].

Chen et al. modified liposomes with PG-10-monostearate, and investigated their pharmacokinetics, in vivo distribution, and capacity to induce anti-PEG IgM. The results showed that, compared with unmodified liposomes, PG-10-monostearate significantly prolonged the blood circulation time of liposomes. Importantly, the biodistribution pattern of PG-10-monostearate-modified liposomes was similar to that of PEGylated liposomes, but they were less likely to induce the production of anti-PEG IgM [99]. Therefore, PGFEs may be a potential good choice to replace PEG for modifying liposomes.

## 4. Conclusions

Polyglycerol fatty acid esters (PGFEs) are a class of nonionic surfactants with a wide HLB value range. In the food field, PGFEs can not only be used to improve the quality of oils/fats (crystallization-adjusters), cream, and chocolate, but can also be used as emulsifiers to prepare emulsions or nanoparticles loaded with bioactive ingredients or flavors to improve their water-solubility, stability, and bioaccessibility. In addition, PGFEs also have antibacterial effects on specific bacteria and are expected to be potential food preservatives. In the field of cosmetics, PGFEs are commonly used as emulsifiers, emulsion stabilizers, cleansing agents, solubilizers, etc. The safety of 274 PGFEs was assessed by the Expert Panel for Cosmetic Ingredient Safety [55], and PGFEs are considered safe for use in cosmetics in their present uses and concentrations, as described in their safety assessment. In the pharmaceutical field, PGFEs can be used for the delivery of drugs and peptides, and can also be used as lipid-based excipients to improve the processing of formulations.

Although PGFEs have a broad range of applications and prospective applications, many problems remain. For example, considering the complexity of polyglycerol, what effects do the polydispersity and branching degree of polyglycerol have on the properties of PGFEs, and how do these further affect the practical applications of PGFEs? The phase behaviors of PGFEs in more mixed systems also require further in-depth research to expand their applications. In addition, the safety of PGFEs in food and cosmetics has been systematically evaluated, but safety assessments regarding their use in pharmaceuticals require more extensive research.

## Figures and Tables

**Table 1:** The chemical structures, HLB values, and uses of PGFEs.

PGFEs	Polyglycerol ^a^	Fatty Acids ^b^	DE ^c^ or FA:PG	HLB	Use	Ref.
PGL6FA10	PG-6	C10	mono-	15.3 ^d^	induce the formation of lecithin reverse wormlike micelles	[18]
PGL10FA6	PG-10	C6	mono-	18.6 ^d^		
PGL10FA10	PG-10	C10	mono-	16.8 ^d^		
PGL10FA14	PG-10	C14	mono-	15.2 ^d^		
PGL10FA18	PG-10	C18	mono-	14.0 ^d^		
PGL20FA10	PG-20	C10	mono-	18.1 ^d^		
PGL40FA10	PG-40	C10	mono-	19.0 ^d^		
M-7D	PG-7	C12	mono-	16	emulsifier (o/w)	[19,20]
M-10D	PG-10	C12	mono-	15		
SWA-10D	PG-10	C18	di-	14		
PG-5 fatty acid esters	PG-5	C6	mono- di- tri- and tetra- hepta-	1.4–16.6	o/w emulsifier (HLB > 9, oil/water = 1:1) w/o emulsifier (HLB < 9, oil/water = 1:1)	[21]
		C8				
		C10				
		C12				
		C14				
		C16				
		C18				
Geomulse^TM^ C15 RSPO MB	PG-10	C8/C10	mono-	15	-	[22]
Geomulse^TM^ L15 RSPO MB		C12	mono-	15		
Geomulse^TM^ S10 RSPO MB		C18	mono-	10		
Geomulse^TM^ O13 RSPO MB		C18:1	mono-	13		
PGE	-	C8-C14/C16-C22 (40/60)	92.8%	4	crystallization-adjuster	[23]
PGEmix-8	-	C16/C18/C18:1	-	1.6	crystallization-adjuster	[24,25]
Witepsol^®^PMF 122	PG-2	C22	Partial	-	lipid-based excipients	[26]
Witepsol^®^PMF 282	PG-2	C18	Full	2.6		
Witepsol^®^PMF 222	PG-2	C22	Full	1.8		
Witepsol^®^PMF 123	PG-3	C22	Partial	-		
Witepsol^®^PMF 1683	PG-3	C16/C18	Partial	5.1		
Witepsol^®^PMF 164	PG-4	C16	Partial	6.0		
Witepsol^®^PMF 1684	PG-4	C16/C18	Partial	5.9		
Witepsol^®^PMF 124	PG-4	C22	Partial	-		
Witepsol^®^PMF 2684	PG-4	C16/C18	Full	3.3		
Witepsol^®^PMF 2686	PG-6	C16/C18	Full	3.1		
PG(2)ME	PG-2	distilled soya acid oil	mono-	6.4	emulsifier (o/w)	[14]
PG(2)DE	PG-2		di-	4.0		
PG(2)TE	PG-2		tri-	2.6		
PG(3)ME	PG-3		mono-	7.9		
PG(3)DE	PG-3		di-	5.6		
PG(3)TE	PG-3		tri-	4.2		
PGO	PG-2.83	C8	mono-	9.4	emulsifier (o/w)	[27]
PGC		C10	mono-	8.9		
PGL		C12	mono-	8.6		
PGM		C14	mono-	8.2		
PGP		C16	mono-	7.5		
PGS		C18	mono-	7.1		
PG-3 monolaurate	PG-3	C12	mono-	8.5	foam stabilizer	[28,29,30]
PG-3 monostearate	PG-3	C18	mono-	6.2		
PG-10 monostearate	PG-10	C18	mono-	11.3		
PGE 55	PG 2-4	C16/C18		7		
PG-10 monolaurate	PG-10	C12	mono-	15.5	emulsifier (o/w)	[31]
PG-10 monomyristate	PG-10	C14	mono-	14.0		
PG-10 monopalmitate	PG-10	C16	mono-	-		
PG-10 monostearate	PG-10	C18	mono-	12.0		
PG-2 monostearate	PG-2	C18	mono-	5.0		
TEGO^®^ Care PSC 3	PG-3	C18 with substoichiometric amount of citric acid	-	~11	emulsifier (o/w)	[32]
TEGOSOFT^®^ PC 41	PG-4	C10	-	~14		[33]
TEGO^®^ Solve 90 MB	PG-6	C8	-	~15		
TEGO Care^®^ PL 4 TEGO^®^ Solve 61 (PG-6 caprylate/PG-3 cocoate/PG-4 caprate/PG-6 ricinoleate = 1/1/1/1)	PG-4 -	C12 -	- -	~11 ~11	emulsifier (o/w)	[34]
PG-4 laurate	PG-4	C10	mono-	10.3	emulsifier (o/w)	[35]
PG-10 monostearate	PG-10	C18	mono-	12.0	niosomes	[36]
PG-10 distearate	PG-10	C18	di-	9.5		
PG-10 tristearate	PG-10	C18	tri-	7.5		
PG-10 diisostearate	PG-10	iso-C18	di-	10.0		
PG-10 caprylates	PG-10	C8	mono-	~14	detergent	[37]

^a^ PG-*n*: PG = polyglycerol; *n* = the average degree of polymerization of PG; ^b^ FA = fatty acid; C6 = hexanoic acid; C8 = caprylic acid; C10 = capric acid; C12 = lauric acid; C16 = palmitic acid; C18 = stearic acid; C18:1 = oleic acid; C22 = behenic acid; ^c^ DE = degree of esterification; ^d^ calculated using organic conceptual diagrams. “-” = Not provided.

## Data Availability

Not applicable.
